# Hypermethylation of lysosomal-associated genes *LAMP1* and *LAMP2* compromises lysosome function in patients with acute lymphoblastic leukemia

**DOI:** 10.1007/s12026-025-09712-8

**Published:** 2025-11-19

**Authors:** Rofaida Refaai, Sara Fouda, Doaa M. Hefni, Dina Ragab, Amany M. Elshamy, Hamada Shoaib, Adel A. Guirgis, Hany Khalil

**Affiliations:** 1 Department of Molecular Biology, Genetic Engineering and Biotechnology Research Institute, University of Sadat City, El-Sadat, Egypt; 2 Department of Clinical Pathology, Faculty of Medicine, Ain Shams University, Cairo, Egypt; 3 Medical Laboratory Science Department, School of Allied Health Sciences, Badr University in Cairo, Badr, Cairo Egypt; 4 Healthcare Department, Saxony Egypt University for Applied Health Sciences, Badr, Cario Egypt; 5 Chemistry Department, Faculty of Science, Mansoura University, Mansoura, Egypt

**Keywords:** Acute lymphocytic leukemia, DNA methylation, LAMP1, LAMP2, TNFα, IL-27

## Abstract

DNA methylation plays a pivotal role in the pathogenesis of Acute Lymphocytic Leukemia (ALL), a hematological malignancy marked by abnormal cellular behavior and immune dysregulation. This study aimed to investigate how alterations in DNA methylation affect lysosmal function in pediatric ALL. A total of 50 blood samples were collected from children diagnosed with ALL and analyzed for biochemical markers associated with the disease. Expression levels of key DNA methylation regulators, including DNA methyltransferase 1 (DNMT1) and DNMT3, were evaluated and compared with those from healthy controls. In addition, pro-inflammatory cytokines, interleukin-6 (IL-6), interleukin-27 (IL-27), and tumor necrosis factor-alpha (TNF-α), were monitored over a six-day period prior to treatment initiation. The study also assessed the expression of lysosome-associated membrane proteins, LAMP1 and LAMP2, which are essential for lysosomal function and the degradation of autophagosomes. To determine the DNA methylation status of the promoter regions of these genes, genomic DNA underwent sodium bisulfite treatment and digestion with methylation-sensitive and methylation-dependent restriction enzymes, followed by amplification with gene-specific primers. Our results revealed a significant upregulation of DNMT1 and DNMT3 in ALL samples, along with a marked downregulation of TET1 gene expression, which is responsible for DNA demethylation. This suggests that disrupted DNA methylation dynamics may contribute to the pathogenesis of the disease. Furthermore, methylation levels within the CpG islands of the LAMP1 and LAMP2 promoter regions were substantially elevated, showing more than a seven-fold increase in ALL samples compared to healthy control blood samples. In ALL samples, the expression levels of LAMP1 and LAMP2 were significantly reduced, may due to promoter region hypermethylation, which contributes to lysosomal dysfunction. In parallel, the expression of autophagy-related genes such as ATG5 and LC3B, markers of autophagy initiation and maturation, respectively, was markedly increased, suggesting an accumulation of autophagosomes that depend on functional lysosomes for complete degradation. Additionally, elevated levels of pro-inflammatory cytokines IL-6, IL-27, and TNF-α were consistently observed in ALL patients, indicating heightened immune activation that may drive disease progression. Collectively, these findings underscore the pivotal role of DNA methylation in disrupting lysosomal function, leading to autophagosome accumulation and impaired recycling of cytoplasmic components.

**Supplementary Information:**

The online version contains supplementary material available at 10.1007/s12026-025-09712-8.

## Introduction

Acute Lymphoblastic Leukaemia (ALL) is a type of cancer that affects white blood cells, characterized by the rapid proliferation of immature lymphocytes in the bone marrow. This uncontrolled growth displaces normal blood cells, leading to various complications and potentially fatal outcomes [1]. ALL is most commonly diagnosed in children aged 2 to 5 and older adults. While the cure rate for children with ALL is approximately 80% globally, adults have a long-term disease-free survival rate of 45–60% [2].

Epigenetics, which studies heritable changes in gene expression without altering the DNA sequence, plays a crucial role in understanding the development of ALL [3]. During cell differentiation, various modifications, such as DNA methylation, histone changes, and nucleosome positioning, occur, enabling cells to maintain distinct identities despite sharing the same DNA [4]. DNA methylation involves the addition of methyl groups to specific regions of DNA, primarily at cytosine residues within CpG dinucleotides [3, 5]. This process is catalyzed by enzymes called DNA methyltransferases (DNMTs), which include DNMT1, DNMT2, DNMT3a, DNMT3b, and DNMT3L. DNMT enzymes establish new methylation patterns on DNA, while “maintenance” DNMTs replicate these patterns onto newly synthesized DNA strands during cell replication [6]. DNA methylation, although not abundant, occurs in specific regions of the mammalian genome, especially in CpG islands found in gene promoters. Methylation of these CpG islands can inhibit gene expression by preventing the binding of transcription factors [7]. In cancer, abnormal activity of DNMTs, particularly DNMT1 and DNMT3, can result in excessive or insufficient DNA methylation. This imbalance can activate oncogenes that promote unchecked cell division or silence tumor suppressor genes responsible for regulating the cell cycle, DNA repair, and cell death [8]. Additionally, these methylation changes can disrupt autophagy by modifying the expression of genes critical for this process, leading to impaired autophagic flux [9].

Autophagy is a cellular process that involves the degradation and recycling of cellular components through the transport of cytoplasmic materials to lysosomes [10]. It is vital for maintaining cellular balance and regulating pathways involved in cell proliferation, differentiation, and disease progression. DNA methylation significantly impacts genes related to autophagy, particularly those involved in autophagosome formation, maturation, and fusion with lysosomes [11]. Key regulators of autophagy include lysosomal-associated membrane proteins (LAMP1 and LAMP2), which play crucial roles in lysosomal biogenesis, autophagosome-lysosome fusion, and autolysosome formation. LAMP1 regulates the acidic environment within lysosomes, while LAMP2 may facilitate the transport of substances into lysosomes [12]. The methylation of LAMP1 and LAMP2 gene promoters is essential for proper autophagy function. When DNA methylation disrupts the expression of these genes, it can impair lysosomal function, hinder autophagosome-lysosome fusion, and compromise autophagic processes [13]. This disruption leads to the accumulation of damaged organelles, reduced protein quality control, and increased cellular stress. Consequently, this can trigger inflammation, oxidative stress, and cell death, contributing to the release of pro-inflammatory cytokines, such as TNF-α [12]. The breakdown of LAMP1 and LAMP2 can activate inflammasomes, leading to the production of TNF-α [14]. This cytokine can promote tumor growth and progression under certain conditions by stimulating the production of other inflammatory cytokines and chemokines, including interleukin-17 (IL-17), IL-6, and IL-27. IL-27, produced by activated antigen-presenting cells like macrophages and dendritic cells, can activate natural killer (NK) cells and CD8 + T cells, enhancing their cytotoxicity against tumor cells [15]. However, IL-27 can also induce the production of immunosuppressive cytokines, such as IL-10, which dampen anti-tumor immune responses. The tumor-promoting effects of IL-27 have been observed in cancers such as ovarian, pancreatic, and breast cancers [16].

This study aimed to investigate the role of DNA methylation changes in ALL-derived samples by assessing the relative expression of DNMT1 and DNMT2. Additionally, we seeked to examine potential methylation alterations in lysosmal-asscoatied genes, including hypermethylation in the promoter regions of LAMP1 and LAMP2. These changes could disrupt autophagic processes and increase the production of inflammatory cytokines that contribute to the development of ALL.

## Materials and methods

### Samples conditions

Fifty blood samples were collected from children diagnosed with Acute Lymphoblastic Leukemia (ALL), while an additional 50 samples were obtained from healthy children, serving as the control group. All patients received appropriate medical care, supervision, and a precise diagnosis of ALL. These blood samples were subsequently analyzed for various indicators of ALL, including white blood cell count, blast cell count, C-reactive protein (CRP) levels, and interleukin-6 (IL-6) levels.

### Counting white blood cells

A 50 µL sample of collected blood was added to 1000 µL of a 2% filtered acetic acid solution in a 12 × 75 labeled tube, resulting in a dilution factor of 20. The acetic acid lysed the red blood cells, leaving the white blood cells (WBCs) and nucleated red blood cells (NRBCs) intact. Methylene blue powder was then added to the diluent to stain the nuclei of the WBCs. A 10 µL aliquot of the diluted blood was placed on each side of the hemocytometer, and the cells were allowed to settle for 2 min. The count was performed using a 10X objective, and all WBCs in the four large corner squares on both sides of the hemocytometer were counted. The total WBC on each side was presented as WBC per microliter.

### Platelet count assay

Platelet counting was performed using the BD Accuri™ C6 flow cytometer. In this method, 100 µL of EDTA-anticoagulated whole blood was labeled with antibodies targeting specific epitopes on the glycoprotein IIb/IIIa complex of both resting and activated platelets (anti-CD 41) at a 1:1000 dilution, followed by incubation at room temperature for one hour. The total sample volume was then adjusted to 500 µL with PBS. The samples were analyzed by flow cytometry at a flow rate of less than 4,000 events per second, which minimizes the need for adjustments related to coincident platelet/RBC or RBC/RBC events. To determine the RBC/platelet ratio, at least 1,000 platelet events and 50,000 RBC events were collected per sample. Events that displayed both RBC scatter signals and platelet fluorescence were considered as RBC-platelet coincidence events. The platelet count was then calculated using this ratio and the RBC concentration of the original blood sample, which was determined by impedance counting [17].

### Immunotyping assay in ALL

Flow cytometry was used to assess CD10 expression as a marker for precursor B-cells in ALL. For each assay, 1 mL of peripheral whole blood was collected in 1.5 mL tubes. Samples were centrifuged at 1500 rpm for 3 min to separate plasma from red blood cells. A volume of 100 µL of plasma was transferred into clean tubes and processed following the manufacturer’s instructions, except for the lysis buffer, which was substituted. Within 1 h of collection, samples were incubated with mouse anti-human CD10-FITC–conjugated primary antibody (332775, BD Biosciences, CA, USA) for 30 min at room temperature in the dark. Following incubation, 400 µL of PBS was added, and the samples were gently inverted before acquisition on the Accuri C6 flow cytometer [18].

### C reactive protein (CRP) assay

For the CRC detection protocol, an ELISA assay (SEA821Hu, Cloud-Clone Corp, USA) was conducted using blood serum, which was separated from the collected samples using standard laboratory methods with serum-separator Vacutainers. One milliliter of each sample underwent cooling centrifugation for 20 min at 10,000 × g to clarify any lipemic specimens. The resulting suspension was then sonicated with an ultrasonic cell disrupter until the solution became clear. Afterward, the homogenates were centrifuged for 5 min at 10,000 × g, and the supernatant was collected. The cells were precipitated by cooling centrifugation at 10,000 × g for 10 min and washed with PBS under the same centrifugation conditions. The cells were then resuspended in fresh lysis buffer at a concentration of approximately 10⁷ cells/mL. The lysed cells were subsequently centrifuged at 1,500 × g for 10 min at 4 °C to remove cellular debris, then resuspended in 500 µL PBS. Finally, the samples were loaded into an ELISA reader in four replicates. By using different dilutions of standard reagents, the final concentration of CRP was determined based on the standard curve, with measurements taken at a wavelength of 450 nm [19].

### Enzyme-linked immunosorbent assay (ELISA)

The ELISA assay was used to measure the levels of IL-6, IL-27, and tumor necrosis factor alpha (TNF-α) by employing human ELISA kits from Invitrogen (USA). A 100 µL sample was added to each well of an ELISA plate and incubated for 2 h at room temperature (RT) with 100 µL of the control solution and 50 µL of a 1X biotinylated antibody. Following this incubation, 100 µL of a 1X streptavidin-HRP solution was added to each well and incubated in the dark for 30 min. Next, 100 µL of the TMB chromogen substrate solution was added, and the incubation continued for 15 min at RT, protected from light. The reaction was then stopped by adding 100 µL of stop solution. Absorbance was measured at 570 nm for each sample [20, 21].

### DNA isolation and methylation analysis

Genomic DNA was extracted from blood samples of ALL patients and healthy children using a DNA isolation kit (Qiagen, USA). Methylation changes in the promoter regions of LAMP1 and LAMP2 were analyzed in all samples using a sodium bisulfite conversion method. Specific primers were designed for each promoter region: wild type primers that detect methylated regions and modified primers that detect unmethylated regions. One microgram of purified DNA was treated with 1 M sodium bisulfite and 10 µL of DNA-protecting buffer, bringing the total volume to 25 µL, using RNase- and DNase-free water. The mixture was denatured at 95 °C for 5 minutes, then incubated at 50 °C for 7 hours to convert unmethylated cytosines to uracil. This conversion process was repeated three times using a thermal cycler (BIO-RAD, USA) to ensure complete conversion. The treated DNA was then used for conventional PCR amplification of the promoter regions, employing specific primers to detect both methylated and unmethylated fragments. The wild type primers used were: LAMP1-F-5’-AACGCCAGCCCTTGGCGCCCGC-3’ and LAMP1-R-5’-ATGGCGCGAGGCGGCCGGGTACG-3’. The modified primers used were: LAMP1-M-F-5’-ACGCCAGCCCTTGGCGCCC-3’ and LAMP1-M-R-5’-ATGGAGCGAGGAGGCAGGGT-3’. The PCR conditions were as follows: an initial denaturation at 95 °C for 10 min, followed by 40 cycles of 95 °C for 30 s, 60 °C for 30 s, and 72 °C for 45 s. The PCR products were separated on a 1% agarose gel in 1X-TBE buffer, and visualized under UV light (320 nm) using a gel documentation system. Methylated fragments appeared as a single band at 80 bp, while unmethylated fragments were represented as a single band at 90 bp [4, 22].

To further evaluate the methylation status of the promoter regions in the selected genes, the genomic DNA was subjected to digestion with the methylation-sensitive restriction enzyme HpaII. HpaII cleaves unmethylated cytosine residues within its restriction site (CCGG). The digestion was performed using 5 units of enzyme per 200 ng of DNA for 4 h at 37 °C. After digestion, the DNA was used for quantitative RT-PCR (qRT-PCR) analysis of the LAMP1 and LAMP2 gene promoter regions, employing the previous wild-type specific primers for each region. The qRT-PCR was carried out using the QuantiTect SYBR Green PCR Kit (Qiagen, USA), with GAPDH promoter primers as a housekeeping gene for normalization. The PCR conditions were as follows: an initial denaturation at 95 °C for 5 min, followed by 35 cycles of 95 °C for 30 s, 60 °C for 30 s, and 72 °C for 30 s, with a final extension at 72 °C for 10 min to complete the amplification [5, 23].

### qRT-PCR procedure for gene expression

Total RNA was extracted from the derived samples using the TriZol reagent (Invitrogen, USA), along with chloroform and isopropanol, following the standard protocol. The extracted RNA was then purified using an RNA purification kit (Invitrogen, USA) according to the manufacturer’s instructions. The purified RNA was utilized to synthesize complementary DNA (cDNA) using the QuantiTech Reverse Transcription Kit (Qiagen, USA) following the provided protocol. The relative expression levels of DNMT1, DNMT3, methionine synthases (MS), ten-eleven translocation (TET1), LAMP1, LAMP2, ATG5, and LC3B were quantified in patient samples and normalized to their expression in control samples using specific primers (refer to Table 1). PCR amplification of the targeted gene fragments was carried out using the QuantiTect SYBR Green PCR Kit (Qiagen, USA), along with GAPDH, which served as the housekeeping gene control. The PCR machine was programmed with the following conditions: 94 °C for 5 min, followed by 40 cycles of 94 °C for 30 s, 60 °C for 30 s, and 72 °C for 45 s. The relative expression of each gene was calculated by normalizing the cycle threshold (Ct) value of each target gene to GAPDH and adjusting between patient and control samples. The relative gene expression levels were then determined using the delta-delta Ct method and are presented as fold changes [24, 25].

**Table 1:** Oligonucleotide sequences employed for the quantitative analysis of the specified genes using qRT-PCR

Description	Primer sequences 5’−3’
DNMT1-sense	CCCATGCATAGGTTCACTTCCTTC
DNMT1-antisense	TGGCTTCGTCGTAACTCTCTACCT
DNMT3-sense	TGCAATGACCTCTCCATTGTCAAC
DNMT3-antsense	GGTAGAACTCAAAGAAGAGGCGG
MS- sense	GAGATGCCTGAGACACCCA
MS- antisense	GTGCACCAGTTTTCGTTCCT
TET1- sense	GCACGATGCACCTGTACGAT
TET1- antisense	CACCAAGCTTTTTTGCTGTGAGT
LAMP1- sense	GTTTCTTCATTCTTTACTG
LAMP1- antisense	TCTCTACTGTTGTAATGT
LAMP2- sense	GCAGTGCAGATGAAGACAAC
LAMP2- antisense	AGTATGATGGCGCTTGAGAC
ATG5-sense	AAAGATGTGCTTCGAGATGTGT
ATG5-antisense	CACTTTGTCAGTTACCAACGTCA
LC3B-sense	AGAGTCGGATTCGCCGCCGCA
LC3B-antisense	GACGGCATGGTGCAGGGATCT
GAPDH-sense	TGGCATTGTGGAAGGGCTCA
GAPDH-antisense	TGGATGCAGGGATGATGTTCT

### Flow cytometric protein profiling

To assess the kinetic expression of LAMP1, LAMP2, ATG5, and LC3B in the collected samples, a flow cytometry-based analysis was performed. Initially, blood samples were centrifuged at 1500 rpm for 3 min to separate plasma from red blood cells. The plasma was then transferred to clean tubes and subjected to a second centrifugation at 1500 ×g for 5 min. Following this, the supernatant was discarded, and the resulting cell pellet was washed with phosphate-buffered saline (PBS), centrifuged again, and subsequently fixed in 2% formaldehyde prepared in PBS. For cell permeabilization, the fixed cells were incubated for 3 min in PBS containing 0.1% Triton X-100. Primary antibody staining was carried out by incubating the permeabilized cells overnight at 4 °C in PBS with 1% bovine serum albumin (BSA). The primary antibodies used were: rabbit monoclonal anti-LAMP1 (MA5-29385, ThermoFisher Scientific, USA), rabbit polyclonal anti-LAMP2 (BS-2379R, ThermoFisher Scientific, USA), and rabbit polyclonal anti-LC3B (ab51520, Abcam, USA), each applied at a 1:100 dilution. After overnight incubation, cells were washed and then incubated for 2 h at room temperature with a secondary antibody, Alexa Fluor 488-conjugated goat anti-rabbit IgG (ab150077, Abcam, USA), diluted 1:500 in PBS containing 1% BSA. Finally, the cells were resuspended in 1 mL of PBS and analyzed using a BD Accuri C6 Plus flow cytometer. Protein expression levels were detected using the FITC channel. LAMP1 and LAMP2 signals primarily appeared in the lower-right quadrant of the flow cytometry plots, represented by blue fluorescence. Similarly, LC3B expression was detected using the same fluorescence settings and visualized as red signals in the corresponding quadrant. Cells with low fluorescence intensity consistently appeared in the lower-left quadrant, indicating low or negligible expression of the target proteins [26, 27].

### Statistical analysis

Delta-delta Ct analysis was used to evaluate relative gene expression, represented as fold changes in steady-state mRNA levels. Ct values for the housekeeping gene GAPDH served as the reference for normalization. A two-tailed t-test was conducted to assess differences in the data, with *P* < 0.05 indicating statistical significance (*) and *P* < 0.01 denoting high statistical significance [28, 29].

## Results

### Biochemical assessment and confirmation of ALL in the collected samples

**Fig. 1:**
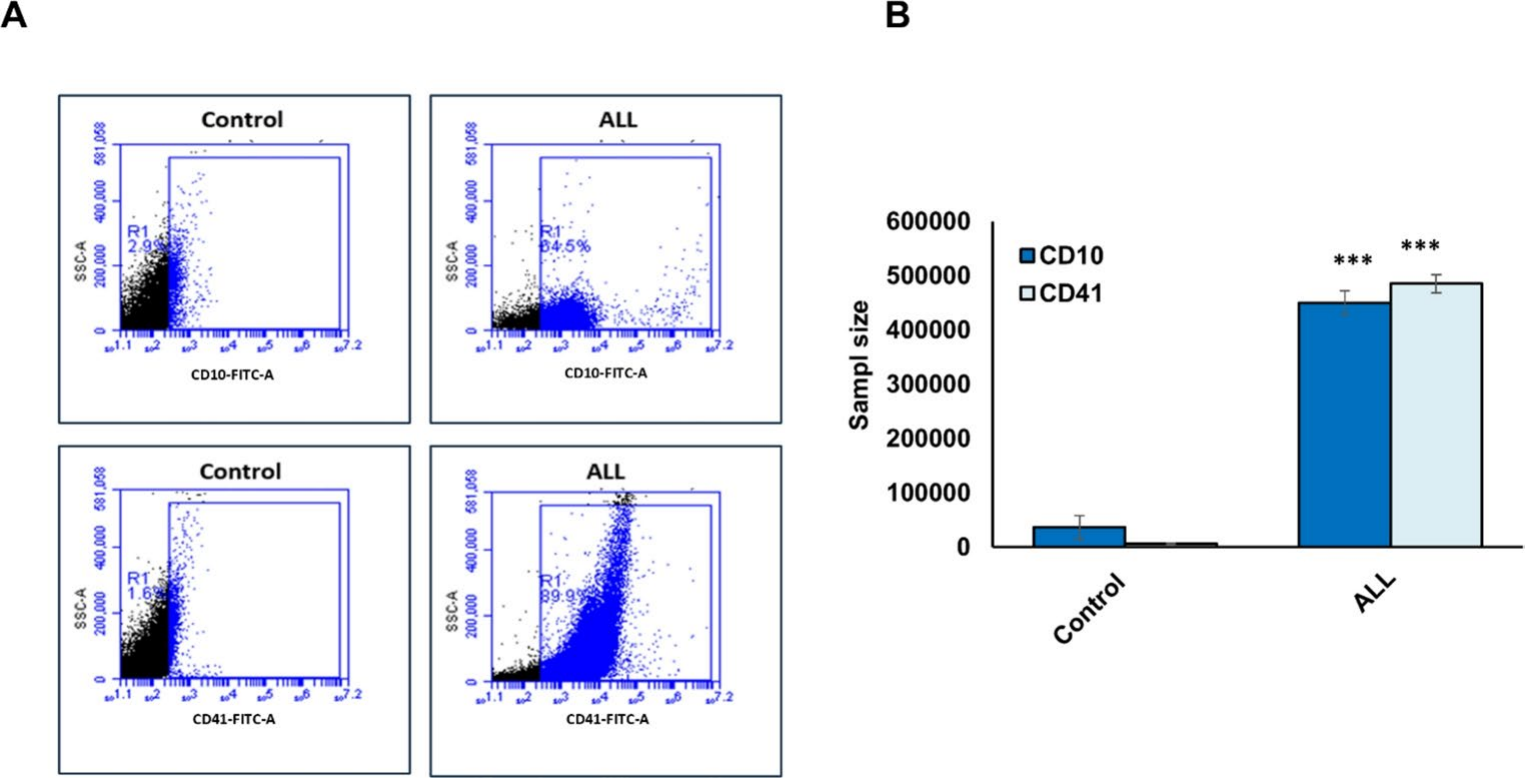
Flow cytometry analysis of ALL cell surface antigen and activated platelets. (**A**) Representative flow cytometry plots showing the proportion of CD10-positive cells (precursor B-cell antigen) and CD41-positive cells (activated platelets) in blood samples from ALL patients compared with healthy controls. (**B**) Mean counts of CD10- and CD41-positive cells from a total of 500,000 analyzed cells per group in ALL patients versus healthy individuals. Error bars represent the standard deviation (SD) from 50 independent blood samples per group. Statistical significance was determined using a two-tailed Student’s *t*-test. Asterisks (***) indicate *P* ≤ 0.001. Data are based on analyses of 50 blood samples from ALL patients and 50 from healthy controls

Flow cytometry was used to assess and diagnose ALL by quantifying the expression of leukemic cell surface antigen and activated platelets in peripheral blood samples. Cells were stained with antibodies against CD10, a hallmark precursor B-cell antigen, and CD41, a marker of platelet activation. CD10 expression was significantly elevated, with approximately 65% of cells staining positive (Fig. 1A, blue). Likewise, CD41 expression was markedly increased in ALL samples, with nearly 89% positivity (Fig. 1A, blue). In absolute terms, more than 400,000 of the 500,000 analyzed cells were CD10-positive, while approximately 450,000 of 500,000 cells expressed CD41. Both markers demonstrated pronounced upregulation compared with healthy control samples (Fig. 1B). Hematological parameters were also compared between healthy individuals and ALL patients (Table 2). White blood cell (WBC) counts were markedly elevated in ALL patients, with mean values of 95,000 cells/mL, compared to approximately 6,500 cells/mL in healthy individuals. This disparity suggests that WBC counts could serve as a valuable diagnostic marker for distinguishing ALL patients from healthy controls. Likewise, platelet counts were significantly increased in ALL samples, averaging 250,000/mL, whereas healthy individuals had mean platelet counts of about 100,000/mL. In addition, inflammatory markers showed notable elevations: CRP levels averaged 15 mg/L in ALL patients compared to 5 mg/L in healthy individuals, while IL-6 concentrations reached 250 pg/mL in ALL patients versus 50 pg/mL in controls. Collectively, these results reveal distinct hematological and immunological alterations between ALL patients and healthy individuals, highlighting the diagnostic and prognostic relevance of flow cytometry findings, WBC, platelet counts, and inflammatory biomarkers in ALL.

**Table 2:** Comparison of demographic variables, WRC counts, platelet count, CRP, and IL-6 production in ALL-derived samples versus healthy individuals

Factor Case	White blood cells count/mL		Platelet count/mL		CRP mg/L		Mean concentration of IL-6 pg/mL	
	Mean value	*P* value	Mean values	*P* value	Mean value	*P* value	Mean value	*P* value
Healthy (*n* = 50)	6500		100,000		7.5		50	
ALL (*n* = 50)	95,000**	0.001	250,000**	0.01	15*	0.02	250**	0.001

### The relative expression of DNA methylation co-factors in children with ALL

**Fig. 2:**
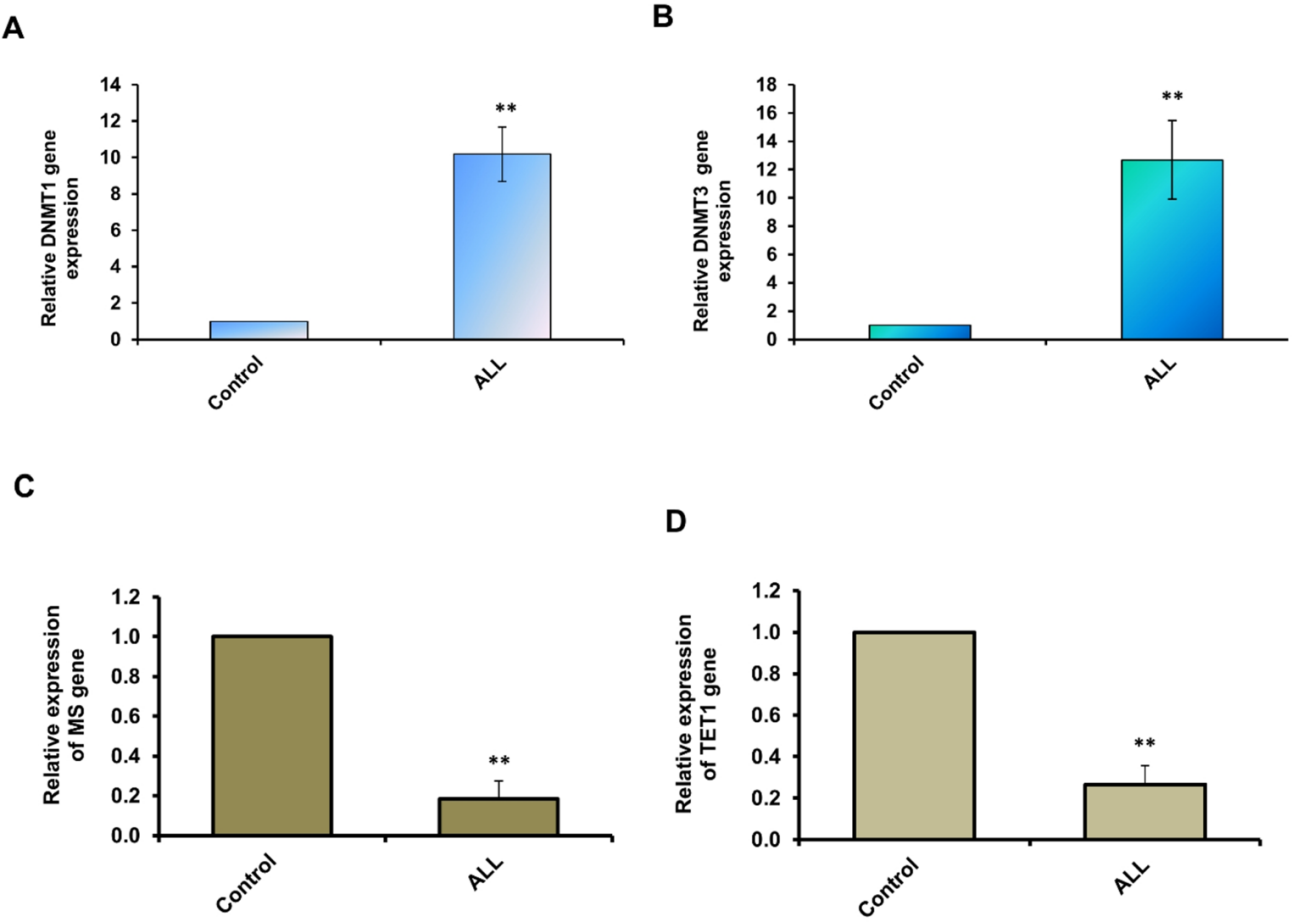
Relative expression levels of DNA methylation–related factors in ALL. (**A**) Relative expression of DNMT1 in samples from ALL patients compared with healthy controls, measured by qRT-PCR. (**B**) Fold-change expression of DNMT3a in ALL patient samples compared with controls. (**C**) Relative expression of MS in ALL patients versus healthy individuals. (**D**) Fold-change expression of TET1 in ALL patients compared with controls. Error bars represent the standard deviation (SD) from three independent experiments. Statistical significance was determined using a two-tailed Student’s *t*-test on Ct values. Asterisks (**) indicate *P* ≤ 0.01. Data are based on the analysis of blood samples from 50 ALL patients and 50 healthy individuals

This study aimed to investigate the influence of DNA methylation in acute lymphoblastic leukemia (ALL) by analyzing the expression of key DNA methylation regulators. Quantitative RT-PCR was used to assess the expression levels of DNA methyltransferases (DNMTs) in blood samples from ALL patients and healthy controls. Our results demonstrated a marked upregulation of both DNMT1 and DNMT3a in ALL patients. Specifically, DNMT1 expression showed nearly a 10-fold increase in patient samples compared to controls (Fig. 1A; Supp. Table 1). Similarly, DNMT3a was significantly elevated, with more than a 10-fold increase relative to healthy individuals (Fig. 2B; Supp. Table 2). These findings indicate that DNMT overexpression may contribute to aberrant DNA methylation in ALL. To further evaluate methylation-related pathways, we examined the expression of MS and TET1. In contrast to DNMTs, MS was markedly downregulated, with approximately a 5-fold reduction in the ALL group (Fig. 2C; Supp. Table 3), suggesting impaired methionine regeneration, an essential step for sustaining DNA methylation. Likewise, TET1 expression was reduced by nearly 4-fold in ALL patients compared to controls (Fig. 2D; Supp. Table 4), implying decreased DNA demethylation activity. Taken together, these results highlight significant dysregulation of both methylation and demethylation factors in ALL. Overexpression of DNMT1 and DNMT3a, coupled with suppression of MS and TET1, may drive the aberrant epigenetic modifications associated with leukemia development.

### Methylomic changes in the LAMP1 and LAMP2 promoter regions

**Fig. 3:**
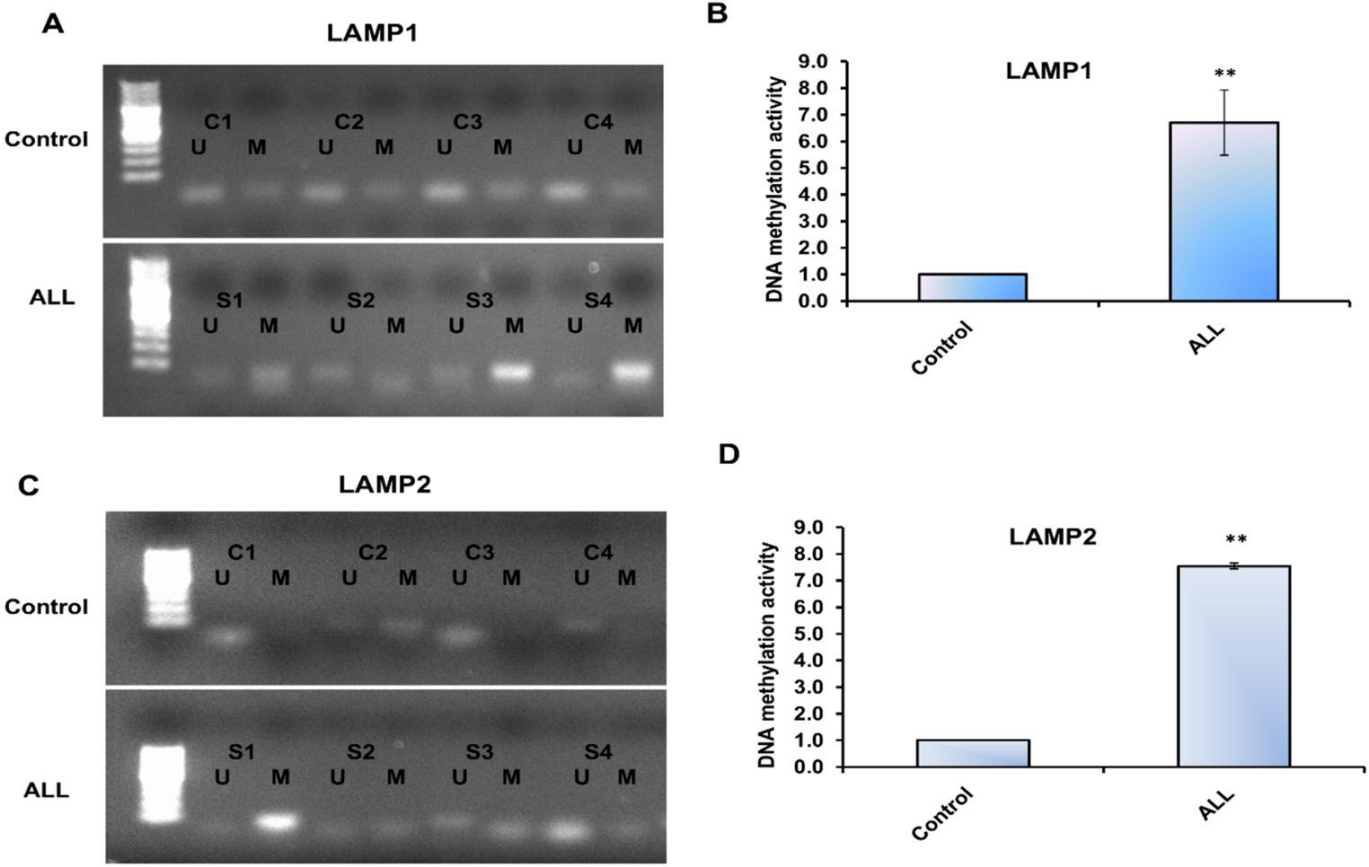
Methylation processes occurring in the promoter regions of LAMP genes. (**A**) Agarose gel electrophoresis displays the amplified genomic DNA fragments from either healthy individuals (upper gel) or patients with acute lymphoblastic leukemia (ALL) (lower gel), following treatment with sodium bisulfite and amplification using specific wild-type or modified primers targeting the LAMP1 promoter region via conventional PCR. (**B**) Methylation activity in the LAMP1 promoter region, as assessed by amplifying the HpaII-digested genomic DNA with the wild-type primer in qRT-PCR. (**C**) Agarose gel electrophoresis shows the amplified genomic DNA fragments from either healthy individuals (upper gel) or ALL patients (lower gel), following sodium bisulfite treatment and amplification with specific wild-type or modified primers targeting the LAMP2 promoter region via conventional PCR. (**D**) Methylation activity in the LAMP2 promoter region, evaluated by amplifying the HpaII-digested genomic DNA with the wild-type primer using qRT-PCR. Error bars represent the SD from three independent experiments. Statistical significance of Ct values between different groups was assessed using a two-tailed Student’s t-test, with (**) indicating *P* ≤ 0.01. The data represent analysis of 50 digested blood samples from ALL patients and 50 blood samples from healthy individuals

We next investigated DNA methylation patterns in the promoter regions of LAMP1 and LAMP2 in samples from ALL patients compared with healthy controls. Sodium bisulfite–treated DNA was amplified using both wild-type and bisulfite-modified primers targeting the promoter regions of the LAMP gene family. Bisulfite treatment converts unmethylated cytosines to uracil, while methylated cytosines remain unchanged, enabling discrimination between methylated and unmethylated alleles. Following PCR amplification, distinct fragment sizes were detected on agarose gels: methylated fragments appeared as a single band at 80 bp, while unmethylated fragments appeared at 90 bp. As shown in Fig. 3A and Supp. Figure 1, methylated fragments within the LAMP1 promoter were more abundant in ALL samples than in controls, whereas unmethylated fragments were less amplified in patient samples. Consistently, genomic DNA digested with the methylation-sensitive restriction enzyme HpaII and amplified with wild-type primers revealed a significant 6-fold increase in LAMP1 promoter methylation in ALL patients compared to controls (Fig. 3B). Similarly, increased methylation was observed in the LAMP2 promoter region in ALL samples (Fig. 3C; Supp. Figure 2). Reduced amplification of unmethylated fragments further supported this hypermethylation pattern. Moreover, HpaII digestion followed by amplification with wild-type primers showed a marked 7-fold increase in LAMP2 promoter methylation in ALL samples relative to controls (Fig. 3D). Together, these findings indicate a clear increase in DNA methylation activity within the promoter regions of LAMP1 and LAMP2, which may contribute to altered gene regulation and leukemogenesis in ALL.

### Disturbance of lysosomal-associated gene expression accompanied by increased expression of autophagy-related genes in ALL

**Fig. 4:**
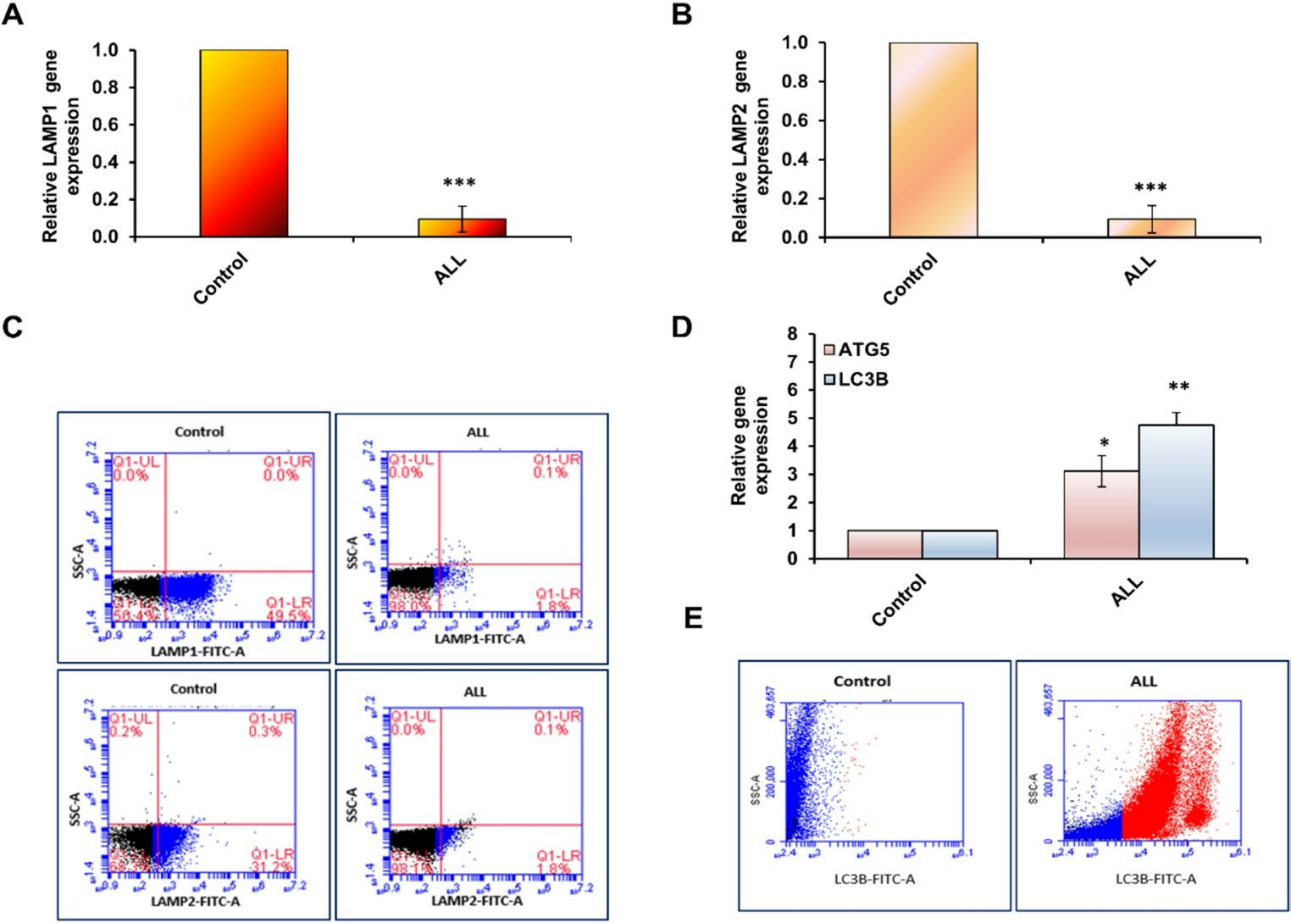
Relative expression levels of lysosomal and autophagy-related genes in ALL. (**A**–**B**) Relative expression of LAMP1 and LAMP2 in blood samples from ALL patients and healthy controls, measured by qRT-PCR. (**C**) Representative flow cytometry plots showing the proportion of LAMP1- and LAMP2-positive cells in ALL patient samples compared with controls. (**D**) Fold-change expression of the autophagy-related genes ATG5 and LC3B in ALL patient samples compared with controls, measured by qRT-PCR. (**E**) Representative flow cytometry plots showing the proportion of LC3B-positive cells in ALL patient samples compared with healthy controls. Error bars indicate the SD from three independent experiments. Statistical significance was determined using a two-tailed Student’s *t*-test on Ct values. Asterisks (***) indicate *P* ≤ 0.001. Data are based on analyses of blood samples from 50 ALL patients and 50 healthy individuals

Lysosomes play a crucial role in the autophagy process, as the fusion of lysosomal vesicles with autophagosomes is essential for the degradation of cellular components. To investigate whether DNA methylation contributes to the regulation of lysosomal-associated and autophagy-related genes, we examined the expression profiles of LAMP1, LAMP2, ATG5, and LC3B using qRT-PCR and flow cytometry. qRT-PCR analysis revealed a significant downregulation of both LAMP1 and LAMP2 in ALL patient samples compared with healthy controls (Fig. 4A and B; Supp. Tables 5–6). This reduction suggests decreased LAMP activity, potentially linked to promoter hypermethylation. Consistently, flow cytometric analysis confirmed reduced protein expression: in ALL samples, only ~ 2% of cells stained positive for LAMP1 compared with ~ 50% of control cells (blue gating). Similarly, LAMP2 expression was markedly reduced, with only ~ 2% positivity observed in patient samples (Fig. 3C). In contrast, the expression of autophagy-related genes was significantly upregulated. qRT-PCR showed a 3-fold increase in ATG5 and a 5-fold increase in LC3B expression in ALL samples relative to controls (Fig. 4D; Supp. Tables 7–8). Flow cytometry further supported these findings, revealing a pronounced increase in LC3B protein expression in ALL-derived cells compared with healthy controls (red gating) (Fig. 4E). Together, these results demonstrate a striking reduction in LAMP1 and LAMP2 expression, accompanied by a strong induction of ATG5 and LC3B in ALL samples. This imbalance suggests a disruption in lysosome–autophagosome fusion and highlights a possible disturbance of the autophagic machinery in leukemia.

### Association between lysosomal dysfunction and cytokine production in ALL

Previous studies have suggested a potential link between lysosomal depletion and cytokine production. To further explore this relationship in the context of ALL, we measured the levels of IL-27 and TNF-α, two cytokines implicated in inflammatory and necrotic processes in blood cells.

**Fig. 5:**
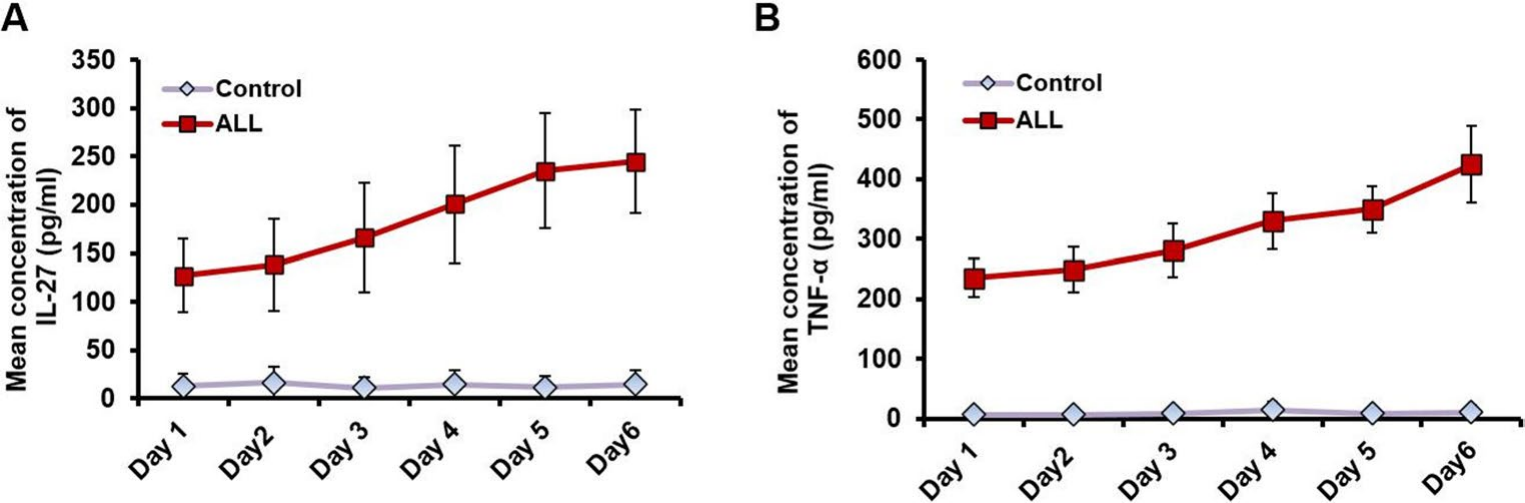
The levels of IL-27 and TNF-α in samples derived from children with ALL. (**A**) The concentration of IL-27 produced (measured in pg/mL) in children with ALL during hospitalization, compared to its concentration in the blood samples of healthy individuals. (**B**) The concentration of TNF-α produced (measured in pg/mL) in children with ALL during hospitalization, compared to its concentration in the blood samples of healthy individuals. Error bars represent the SD of four independent replicates. The data analysis includes 50 patients and 50 healthy individuals

As shown in Fig. 5A, IL-27 concentrations in blood samples from ALL patients increased progressively during hospitalization, exceeding 250 pg/mL by day 6. Similarly, Fig. 5B demonstrates a significant rise in TNF-α levels, which reached nearly 400 pg/mL over the same period. In contrast, cytokine concentrations in healthy individuals remained stable and close to baseline, with values approaching zero throughout the six-day observation period. This reflects the steady, basal expression of TNF-α typically associated with a functional immune system. The pronounced elevation of IL-27 and TNF-α in ALL patients suggests that cytokine dysregulation may be driven, at least in part, by lysosomal dysfunction. Collectively, these findings underscore a potential connection between impaired lysosomal activity and enhanced proinflammatory signaling in ALL pathogenesis.

## Discussion

In this study, hematological parameters, including WBC count, platelet count and activity, CRP, and IL-6 levels, exhibited distinct patterns between the two groups. In addition, CD10, a common ALL-associated antigen, and CD41, a platelet glycoprotein receptor indicative of platelet activation, were evaluated in ALL-derived samples. CD10 is a well-established biomarker in ALL, particularly in precursor B-cell subtypes, where its expression is associated with leukemic cells and a more favorable prognosis [30]. CD41, also known as integrin alpha-IIb, is a platelet-specific protein expressed on activated platelets, and its detection serves as a marker of platelet activation [31]. This study identified significant differences in hematological parameters between healthy individuals and ALL patients. WBC and platelet counts were largely within the normal range in healthy individuals, whereas ALL patients showed significant increase in WBC and platelet count, CRP, and IL-6, underscoring their diagnostic and prognostic relevance.

DNA methylation plays a pivotal role in the development and progression of ALL [32]. Specific DNA methyltransferases (DNMTs) regulate DNA methylation patterns, which significantly influence various cellular processes, including autophagy. Aberrant DNA methylation and hypermethylation can disrupt the normal regulation of autophagy, controlling either its activation or suppression [33]. Several studies have highlighted disease-associated DNA methylation changes linked to autophagy dysfunction in conditions like cancer and neurological disorders [34, 35]. This study further examined DNA methylation regulators (DNMTs) and their interaction with lysosomal-associated genes, particularly LAMPs, in ALL pathogenesis. DNMT1 and DNMT3a were overexpressed in ALL samples, indicating enhanced methylation activity, while MS, a critical methylation cofactor, was consistently downregulated in patient-derived samples. Reduced MS protein activity results in lower methionine levels, a key cofactor for DNA (cytosine-5)-methyltransferase 1 (DNMT1). Since methionine is a precursor for SAMe metabolism, reduced levels may alter DNA methylation [36]. To investigate potential drivers of DNA hypermethylation in ALL, we analyzed TET1, a crucial demethylating enzyme with tumor suppressive functions. We observed reduced TET1 expression, which may impair demethylation, thereby contributing to hematological malignancies such as AML, ALL, MDS, and CML [37]. As TET1 oxidizes 5-methylcytosines to promote DNA demethylation, its downregulation may underlie malignant cell hypermethylation, likely through epigenetic modifications in its promoter region. Prior studies also highlight the role of TET proteins in cancer prevention via regulation of oncogenes, tumor suppressors, and lysosomal-related genes [38, 39]. LAMP1 and LAMP2 play crucial roles in the fusion of autophagosomes with lysosomes, aiding in the degradation of autophagic cargo [10]. They help form the fusion pore, facilitating the exchange of contents between these two organelles.

**Fig. 6:**
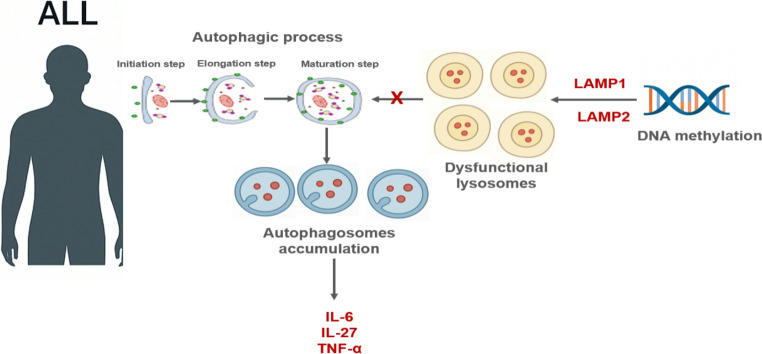
The interplay between methylomic alterations in lysosomal genes, autophagy, and interleukins production in ALL

Based on these findings, we further evaluated LAMP1 and LAMP2 expression and investigated their promoter methylation activity, providing insights into the potential link between DNA methylation and lysosomal dysfunction in relation to autophagy and degradation processes in ALL. Our results revealed an increased level of CpG methylation within the promoter regions of the LAMP1 and LAMP2 genes, which was accompanied by reduced expression of both genes at the RNA and protein levels in ALL samples. This downregulation of LAMP1 and LAMP2 impaired lysosomal function, particularly affecting the fusion and degradation of autophagosomes in patient blood samples. Consequently, the disruption of autophagosome fusion and clearance led to their accumulation, which in turn triggered enhanced necrotic and inflammatory responses, as evidenced by elevated levels of IL-6, IL-27, and TNF-α (Fig. 6). The disruption of LAMP-1 and LAMP-2 genes leads to lysosomal membrane permeabilization (LMP), which results in the release of lysosomal enzymes and reactive oxygen species (ROS) [40]. These factors can activate various inflammatory pathways, including the NLRP3 inflammasome, NF-κB signaling, and the production of pro-inflammatory cytokines such as TNF-α, IL-1β, IL-6, IL-17, and IL-27.

Accumulating evidence underscores the complex interplay between autophagy and cytokine regulation. Autophagy suppresses inflammasome activation, which occurs in myeloid cells and constitutes a key element of innate immunity [41]. Loss of ATG16L1 function results in elevated secretion of IL-1β and IL-18 [42]. Damaged mitochondria act as potent inflammasome activators by releasing mitochondrial DNA and reactive oxygen species (ROS) [43]. Mitophagy, a selective form of autophagy that eliminates dysfunctional mitochondria, is essential for restraining inflammasome activity [41]. In addition, inflammasomes can undergo p62-mediated selective degradation via autophagy, thereby reducing IL-1β and IL-18 production, while IL-1β itself may be directly degraded within autolysosomes [44]. Cytokines such as IL-6 also display bidirectional regulation with autophagy. In human periodontal ligament (HPDL) fibroblasts, IL-6 expression increased in response to mechanical pressure, an effect intensified by autophagy inhibition [45]. In contrast, IL-6 was shown to suppress starvation-induced autophagy through p-STAT3 signaling to downstream effectors, including Bcl-2 and Beclin1 [46]. Similarly, autophagy protects hepatoma cells from TNF-induced cytotoxicity by blocking caspase-8 activation and the mitochondrial death pathway, while its inhibition elevates TNF levels and caspase-8 activity, suggesting therapeutic potential in TNF-mediated tissue damage [47]. IL-27, an immunomodulatory cytokine with both pro- and anti-inflammatory properties, has recently been implicated in autophagy regulation. Initially, IL-27 was found to suppress IFN-induced autophagy [48]. More recent work demonstrated that IL-27 upregulates DNMT1, thereby inhibiting ERK/p38-driven autophagy [49].

Hypermethylation of the promoter regions of LAMP1 and LAMP2, critical lysosomal-associated proteins, disrupts lysosomal function and impairs fusion with mature autophagic vesicles, leading to autophagosome accumulation in ALL patients. In these patients, autophagy is initiated by the conjugation of ATG5, ATG12, and ATG16, followed by the conversion of cytosolic LC3B to membrane-bound LC3B, which is essential for autophagosome elongation and maturation. However, defective lysosome–autophagosome fusion results in excessive accumulation of autophagic vacuoles, triggering cytotoxic effects and enhancing the production of inflammatory cytokines, including IL-6, IL-27, and TNF-α.

On the other hand, excessive accumulation of autophagosomes, caused by defective fusion with lysosomes, can induce cellular toxicity and promote caspase-8 activation, underscoring the dual role of autophagy in both cell survival and cell death [47, 50, 51]. Our findings reveal increased DNMT1 and DNMT3a expression, suggesting altered DNA methylation patterns in ALL patients. Reduced LAMP1 and LAMP2 levels, together with heightened promoter methylation, indicate lysosomal dysfunction, leading to impaired autophagy and accumulation of autophagosomes. Additionally, elevated TNF-α and IL-27 levels reflect abnormal immune activation and dysregulated cytokine production, which may contribute to tumor growth, immune evasion, or disease progression. These results highlight the potential role of TNF-α and IL-27 in ALL pathogenesis. In conclusion, DNA methylation–mediated lysosomal dysfunction and impaired autophagy may enhance cytotoxicity in ALL, warranting further investigation into therapeutic strategies targeting these pathways.

## Conclusion

DNA methylation plays a pivotal role in the pathogenesis of ALL, a hematological malignancy characterized by abnormal cellular behavior and dysregulated immune responses. This study investigated the impact of DNA methylation alterations on lysosomal gene expression and their downstream effects on lysosomal function and autophagic processes in ALL. Our findings revealed significant changes in DNA methylation–related enzymes, particularly DNMT1, DNMT3, and TET1, indicating that aberrant methylation patterns may contribute to leukemogenesis. Importantly, we observed downregulation of LAMP1 and LAMP2, essential mediators of lysosomal integrity and autophagic degradation. This reduction may result from promoter hypermethylation, which likely impairs transcription factor binding and silences gene expression. Consequently, lysosomal dysfunction disrupts autophagic flux, leading to autophagosome accumulation and triggering inflammatory pathways. Elevated levels of pro-inflammatory cytokines, including TNF-α, IL-6, and IL-27, were detected in ALL patients, reflecting abnormal immune activation and suggesting a potential role in disease progression through tumor growth, immune evasion, or enhanced cytotoxicity. Overall, these results underscore the contribution of DNA methylation–driven lysosomal dysfunction and impaired autophagy in ALL development. A better understanding of these molecular mechanisms may provide new therapeutic opportunities targeting DNA methylation and autophagy in ALL management.

## Supplementary Information

Below is the link to the electronic supplementary material.

Supplementary Material 1 (DOCX 660 KB)

## Data Availability

The data supporting these findings are included in the main manuscript.
