# Supplementary material for: Hypermethylation of lysosomal-associated genes LAMP1 and LAMP2 compromises lysosome function in patients with acute lymphoblastic leukemia
Source: Immunol Res. 2025 Nov 19;73(1):167. doi: 10.1007/s12026-025-09712-8 (PMC12627130; doi:10.1007/s12026-025-09712-8)
Supplement: Supplementary file 1 — Supplementary Material 1 (DOCX 660 KB) [file 12026_2025_9712_MOESM1_ESM.docx]

**Supplementary data**

**Supplementary Table 1:** Mean Ct values of the DNMT1 and GAPDH genes expression in blood samples derived from patients with ALL in comparison with healthy individuals (Control) using qRT-PCR.

| **Samples** | **GAPDH** | | | | **DNMT1** | | | | **Δ**  **Ct1** | | **Δ**  **Ct2** | | **Δ- Δ Ct1** | **Δ- Δ Ct2** | | **Ct1**  **Gene expression** | | **Ct2**  **Gene expression** | **Mean** | **SD** | ***P***  **Value** |
| --- | --- | --- | --- | --- | --- | --- | --- | --- | --- | --- | --- | --- | --- | --- | --- | --- | --- | --- | --- | --- | --- |
|  | **MeanCt1** | | **MeanCt2** | | **MeanCt1** | | **Mean Ct2** | |  |  |  |  |  |  |  |  |  |  |  |  |  |
| Control | | 19.33 | | 20.94 | | 30.14 | | 32.00 | | 10.81 | | 11.51 | 0.00 | | 0.00 | | 1.00 | 1.00 | 1.0 | 0.00 |  |
| **ALL** | | 19.32 | | 18.89 | | 26.64 | | 27.21 | | 7.32 | | 8.32 | -3.49 | | -3.19 | | 11.24 | 9.13 | 10.18** | 1.4 | **0.01** |

| **Samples** | **GAPDH** | | **DNMT3a** | | | **Δ**  **Ct1** | | **Δ**  **Ct2** | **Δ- Δ Ct1** | **Δ- Δ Ct2** | | **Ct1**  **Gene expression** | | **Ct2**  **Gene expression** | **Mean** | **SD** | ***P***  **Value** |
| --- | --- | --- | --- | --- | --- | --- | --- | --- | --- | --- | --- | --- | --- | --- | --- | --- | --- |
|  | **MeanCt1** | **MeanCt2** | **MeanCt1** | **Mean Ct2** | |  |  |  |  |  |  |  |  |  |  |  |  |
| Control | 19.87 | 19.99 | 30.84 | | 31.63 | | 10.97 | 11.6 | 0.00 | | 0.00 | | 1.00 | 1.00 | 1.0 | 0.00 |  |
| **ALL** | 19.32 | 18.89 | 26.64 | | 27.11 | | 7.10 | 8.2 | -3.87 | | -3.42 | | 14.65 | 10.72 | 12.6** | 2.78 | **0.01** |

**Supplementary Table 2:** Mean Ct values of the DNMT3a and GAPDH gene expression in blood samples derived from patients with ALL in comparison with healthy individuals (Control) using qRT-PCR.

**Supplementary Table 3:** Mean Ct values of the MS and GAPDH genes expression in blood samples derived from patients with ALL in comparison with healthy individuals (Control) using qRT-PCR.

| **Samples** | **GAPDH** | | **MS** | | **Δ**  **Ct1** | | **Δ**  **Ct2** | **Δ- Δ Ct1** | **Δ- Δ Ct2** | | **Ct1**  **Gene expression** | | **Ct2**  **Gene expression** | **Mean** | **SD** | ***P***  **Value** |
| --- | --- | --- | --- | --- | --- | --- | --- | --- | --- | --- | --- | --- | --- | --- | --- | --- |
|  | **MeanCt1** | **MeanCt2** | **MeanCt1** | **Mean Ct2** |  |  |  |  |  |  |  |  |  |  |  |  |
| Control | 18.7 | 18.1 | 21.2 | 22.1 | | 2.5 | 4 | 0.00 | | 0.00 | | 1.00 | 1.00 | 1.00 | 0.00 |  |
| **ALL** | 19.5 | 19.8 | 19.9 | 20.2 | | 0.4 | 0.3 | 3.4 | | 3.4 | | 0.3 | 0.1 | 0.2** | 0.17 | 0.01 |

**Supplementary Table 4:** Mean Ct values of the TET1 and GAPDH gene expression in blood samples derived from patients with ALL in comparison with healthy individuals (Control) using qRT-PCR.

| **Samples** | **GAPDH** | | | | **TET1** | | | **Δ**  **Ct1** | | **Δ**  **Ct2** | **Δ- Δ Ct1** | **Δ- Δ Ct2** | | **Ct1**  **Gene expression** | | **Ct2**  **Gene expression** | **Mean** | **SD** | ***P***  **Value** |
| --- | --- | --- | --- | --- | --- | --- | --- | --- | --- | --- | --- | --- | --- | --- | --- | --- | --- | --- | --- |
|  | **MeanCt1** | | **MeanCt2** | | **MeanCt1** | **Mean Ct2** | |  |  |  |  |  |  |  |  |  |  |  |  |
| Control | | 17.7 | | 17.1 | 20.2 | | 21.1 | | 2.5 | 4 | 0.00 | | 0.00 | | 1.00 | 1.00 | 1.00 | 0.00 |  |
| **ALL** | | 20.2 | | 20.53 | 19.93 | | 20.15 | | -0.27 | -0.38 | 3.23 | | 2.72 | | 0.37 | 0.16 | 0.27** | 0.26 | 0.01 |

**
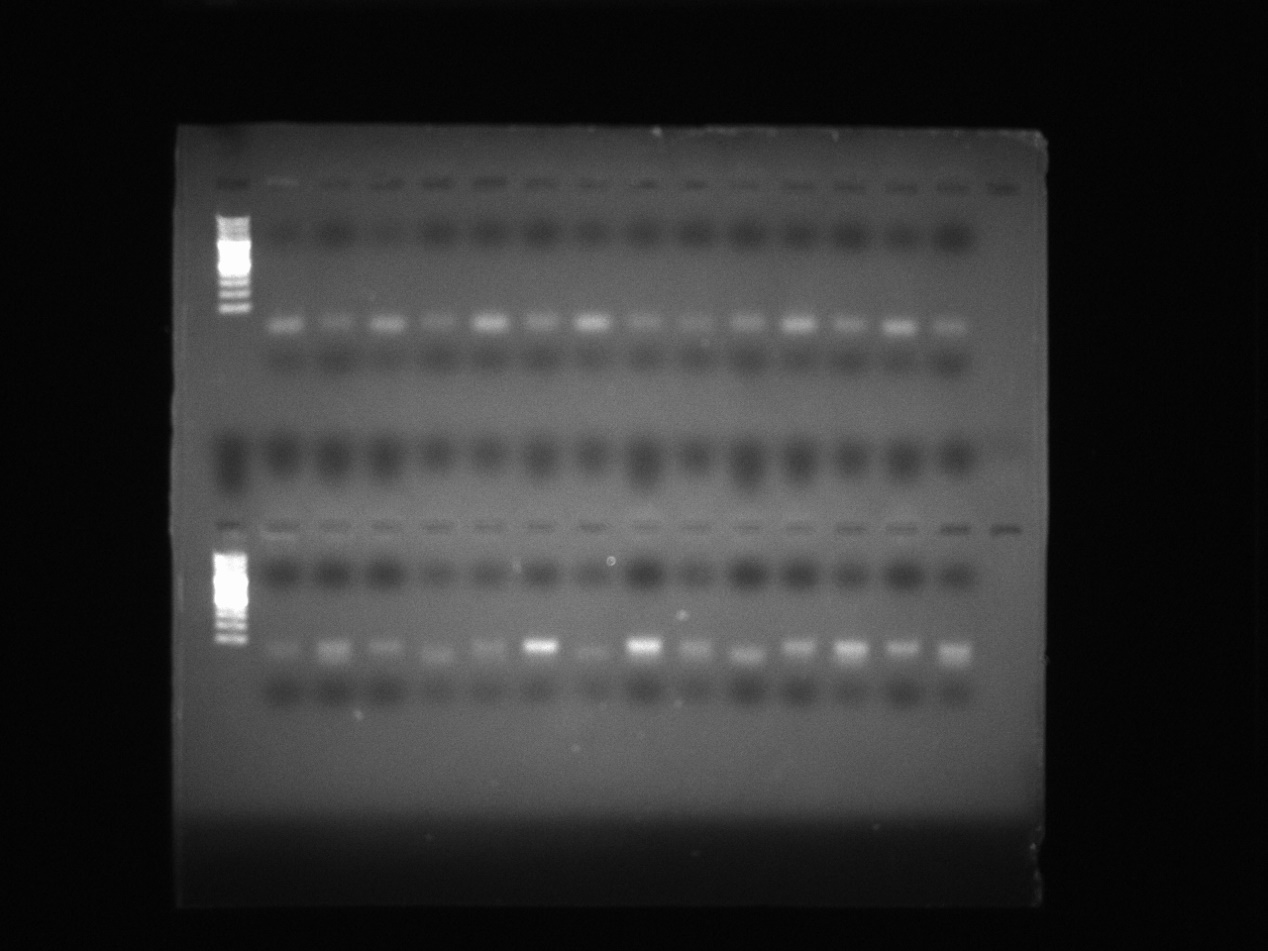
**

**Supp. Figure 1:** The original, uncropped agarose gel displays amplified fragments from genomic DNA isolated from healthy individuals (upper gel) and from ALL patients (lower gel). Prior to amplification, the DNA was treated with sodium bisulfite and then subjected to PCR using either methylation-specific or unmethylation-specific primers targeting the promoter region of LAMP1. A 100 bp molecular weight ladder was included as a size reference.


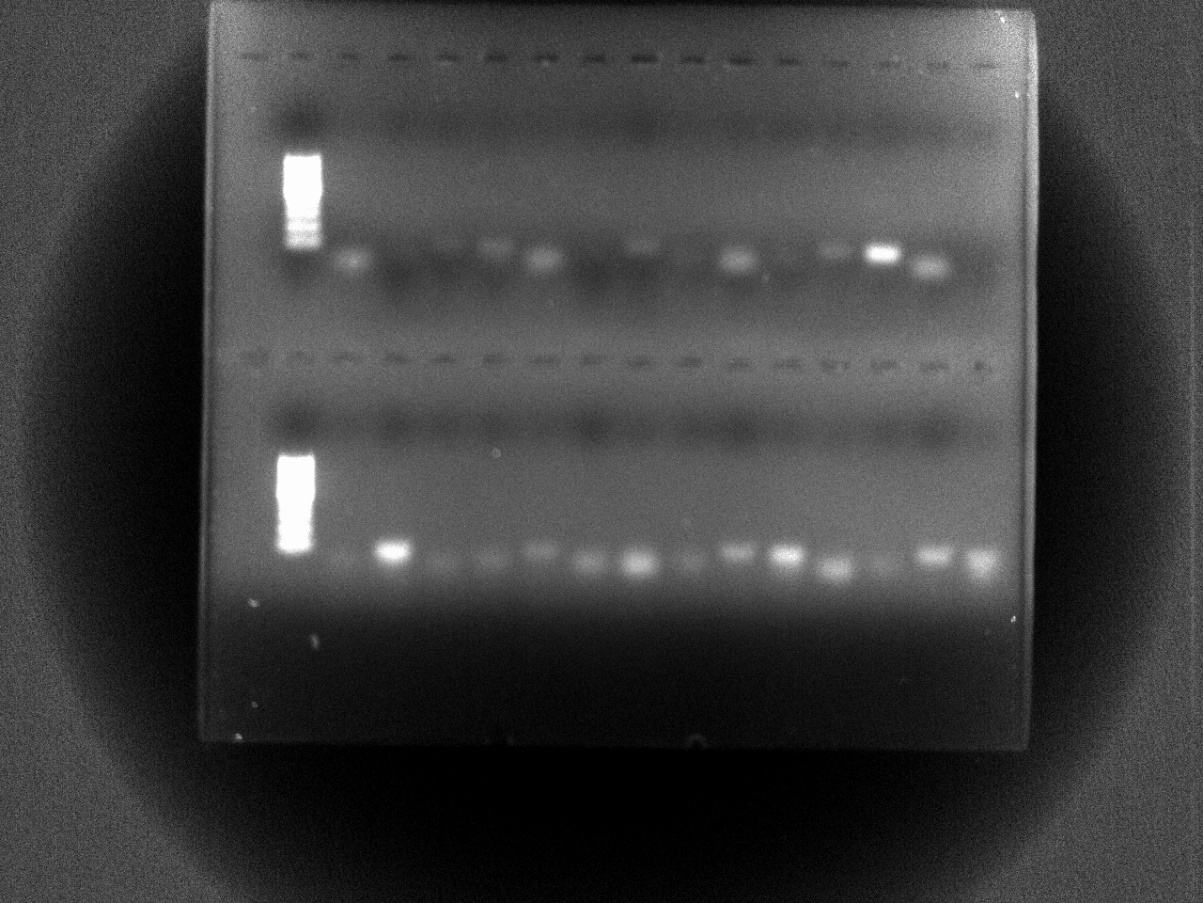


**Supp. Figure 2:** The original, uncropped agarose gel displays amplified fragments from genomic DNA isolated from healthy individuals (upper gel) and from ALL patients (lower gel). Prior to amplification, the DNA was treated with sodium bisulfite and then subjected to PCR using either methylation-specific or unmethylation-specific primers targeting the promoter region of LAMP2. A 100 bp molecular weight ladder was included as a size reference.

.

**Supplementary Table 5:** Mean Ct values of the LAMP1 and GAPDH genes expression in blood samples derived from patients with ALL in comparison with healthy individuals (Control) using qRT-PC

| **Samples** | **GAPDH** | | **LAMP1** | | | **Δ**  **Ct1** | | **Δ**  **Ct2** | **Δ- Δ Ct1** | **Δ- Δ Ct2** | | **Ct1**  **Gene expression** | | **Ct2**  **Gene expression** | **Mean** | **SD** | ***P***  **Value** |
| --- | --- | --- | --- | --- | --- | --- | --- | --- | --- | --- | --- | --- | --- | --- | --- | --- | --- |
|  | **MeanCt1** | **MeanCt2** | **MeanCt1** | **Mean Ct2** | |  |  |  |  |  |  |  |  |  |  |  |  |
| Control | 19.51 | 20.05 | 27.52 | | 27.41 | | 8 | 7..36 | 0.00 | | 0.00 | | 1.00 | 1.00 | 1.0 | 0.00 |  |
| **ALL** | 19.32 | 18.89 | 30.84 | | 31.63 | | 11.52. | 12.7 | 3.51 | | 5.38 | | 0.09 | 0.02 | 0.06*** | 0.05 | **0.0011** |

| **Samples** | **GAPDH** | | **LAMP2** | | | **Δ**  **Ct1** | | **Δ**  **Ct2** | | **Δ- Δ Ct1** | **Δ- Δ Ct2** | | **Ct1**  **Gene expression** | | **Ct2**  **Gene expression** | **Mean** | **SD** | ***P***  **Value** |
| --- | --- | --- | --- | --- | --- | --- | --- | --- | --- | --- | --- | --- | --- | --- | --- | --- | --- | --- |
|  | **MeanCt1** | **MeanCt2** | **MeanCt1** | **Mean Ct2** | |  |  |  |  |  |  |  |  |  |  |  |  |  |
| Control | 19.11 | 19.45 | 27.52 | | 27.41 | | 8 | | 7..36 | 0.00 | | 0.00 | | 1.00 | 1.00 | 1.0 | 0.00 |  |
| **ALL** | 19.13 | 18.79 | 30.64 | | 31.23 | | 11.51. | | 12.44 | 2.80 | | 4.48 | | 0.14 | 0.04 | 0.09*** | 0.07 | **0.0011** |

**Supplementary Table 6:** Mean Ct values of the LAMP2 and GAPDH genes expression in blood samples derived from patients with ALL in comparison with healthy individuals (Control) using qRT-PCR

**Supplementary Table 7:** Mean Ct values and fold changes in the *ATG5* and *GAPDH* gene expression in blood samples obtained from patients with ALL in comparison with healthy individuals using the qRT-PCR

| **Samples** | **GAPDH** | | **ATG5** | | **Δ**  **Ct1** | **Δ**  **Ct2** | **Δ- Δ Ct1** | **Δ- Δ Ct2** | **Ct1**  **Fold change** | **Ct2**  **Fold change** | **Mean**  **Fold change** | **STDV** | ***P***  **Value** |
| --- | --- | --- | --- | --- | --- | --- | --- | --- | --- | --- | --- | --- | --- |
|  | **MeanCt1** | **MeanCt2** | **MeanCt1** | **Mean Ct2** |  |  |  |  |  |  |  |  |  |
| Control | 19.07 | 19.08 | 29.74 | 29.80 | 10.66 | 10.7 | 0.00 | 0.00 | 1.00 | 1.00 | 1.00 | 0.00 |  |
| ALL | 18.92 | 18.89 | 28.14 | 27.81 | 9.22 | 8.92 | -1.45 | -1.81 | 2.72 | 3.50 | 3.11* | 0.55 | **0.03** |

**Supplementary Table 8:** Mean Ct values and fold changes in the *LC3B* and *GAPDH* gene expression in blood samples obtained from patients with ALL in comparison with healthy individuals using the qRT-PCR

| **Samples** | **GAPDH** | | **LC3B** | | **Δ**  **Ct1** | **Δ**  **Ct2** | **Δ- Δ Ct1** | **Δ- Δ Ct2** | **Ct1**  **Fold change** | **Ct2**  **Fold change** | **Mean**  **Fold change** | **STDV** | ***P***  **Value** |
| --- | --- | --- | --- | --- | --- | --- | --- | --- | --- | --- | --- | --- | --- |
|  | **MeanCt1** | **MeanCt2** | **MeanCt1** | **Mean Ct2** |  |  |  |  |  |  |  |  |  |
| Control | 19.07 | 19.08 | 30.43 | 29.63 | 11.36 | 10.6 | 0.00 | 0.00 | 1.00 | 1.00 | 1.00 | 0.00 |  |
| ALL | 18.92 | 19.39 | 28.14 | 27.61 | 9.22 | 8.22 | -2.14 | -2.34 | 4.42 | 5.06 | 4.74** | 0.46 | **0.007** |
